# Draft Genome Sequence of Marine-Derived *Bacillus subtilis* TP-B0611, a Producer of Bacilosarcins and Amicoumacins

**DOI:** 10.1128/genomeA.01134-16

**Published:** 2016-10-13

**Authors:** Hisayuki Komaki, Akira Hosoyama, Natsuko Ichikawa, Yasuhiro Igarashi

**Affiliations:** a Biological Resource Center, National Institute of Technology and Evaluation (NBRC), Chiba, Japan; b NBRC, Tokyo, Japan; c Biotechnology Research Center and Department of Biotechnology, Toyama Prefectural University, Toyama, Japan

## Abstract

Here, we report the draft genome sequence of *Bacillus subtilis* TP-B0611, which produces the isocoumarin-type compounds bacilosarcin and amicoumacin. The genome encodes three nonribosomal peptide synthetase (NRPS) gene clusters and one hybrid polyketide synthase (PKS)/NRPS gene cluster. The hybrid PKS/NRPS gene cluster was identified to be responsible for the biosynthesis of bacilosarcins and amicoumacins.

## GENOME ANNOUNCEMENT

During the course of screening marine-derived bacteria for new bioactive compounds, *Bacillus subtilis* TP-B0611 was isolated from intestine content of a sardine (*Sardinops melanostictus*) collected in Toyama Bay, Japan, and found to produce two new isocoumarin-type compounds, designated bacilosarcins A and B, with herbicidal activity, together with three known isocoumarins, amicoumacins A, B, and C (1). Although bacilosarcins are distinct from amicoumacins due to the presence of 2-hydroxymorpholine moiety, chemical backbones of bacilosarcins and amicoumacins are quite similar. Therefore, these compounds seem to be synthesized by the same pathway. Their chemical structures suggest that they are likely derived from the assembly of polyketide and amino acid units, but their biosynthetic gene clusters had not been reported before we began this study. To identify the gene clusters, we performed a genome analysis of *B. subtilis* TP-B0611.

*B. subtilis* TP-B0611 was deposited in the NBRC culture collection and has been registered as NBRC 110487. A monoisolate of *B. subtilis* TP-B0611 was subjected to a genome sequencing project using a combined strategy of shotgun sequencing with GS FLX+ (Roche; 81.7-Mb sequences, 20.2-fold coverage) and paired-end sequencing with the HiSeq1000 platform (Illumina; 393.3-Mb sequences, 97.4-fold coverage). These reads were assembled using Newbler version 3.0 and subsequently finished using GenoFinisher (2), which led to a final assembly of 14 scaffolds and three contig sequences of >500 bp each. The total size of the assembly was 4,018,468 bp, with a G+C content of 48.3%. Coding sequences were predicted by Prodigal (3). Polyketide synthase (PKS) and nonribosomal peptide synthetase (NRPS) gene clusters were surveyed in the same manner as previously reported (4).

The genome harbored three NRPS gene clusters and one hybrid PKS/NRPS gene cluster, which are encoded in scaffold00003, scaffold00005, scaffold00008, and scaffold00006, respectively. Similarity searches using antiSMASH (5) suggested that three NRPS gene clusters are responsible for syntheses of fengycin, surfactin, and bacillibactin, respectively. During this study, the amicoumacin-biosynthetic gene (*ami*) cluster was identified from *B. subtilis* 1779 (6). Since gene orders and domain organizations are completely identical between the hybrid PKS/NRPS gene cluster of *B. subtilis* TP-B0611 and the *ami* cluster of *B. subtilis* 1779, the hybrid PKS/NRPS gene cluster could be plausibly assigned to the biosynthetic gene cluster for amicoumacins and bacilosarcins. However, the mechanism of formation of the 2-hydroxymorpholine moiety, an unusual cyclic structure in bacilosarcins, cannot at present be elucidated by bioinfomatic analysis only.

Interestingly, BLAST searches suggested that gene clusters similar to amicoumacin/bacilosarcin-biosynthetic gene clusters are also present in other *Bacillus* strains, such as *Bacillus* sp. JS, *Bacillus* sp. A053, *B. subtilis* subsp. *inaquosorum* KCTC 13429, *B. subtilis* subsp. *inaquosorum* DE111, and *B. subtilis* gtP20b. These strains therefore may produce isocoumarin-type compounds such as amicoumacins and bacilosarcins. The genome sequence of *B. subtilis* TP-B0611 will provide useful information on the biosynthetic mechanism of bacilosarcins.

### Accession number(s).

The draft genome sequence of *Bacillus subtilis* TP-B0611 has been deposited in the DDBJ/ENA/GenBank database under the accession number BDFC00000000. The version described in this paper is the first version, BDFC01000000.
